# Do Patients Aged 85 Years and above Benefit from Their Cochlear Implants?

**DOI:** 10.3390/audiolres13010010

**Published:** 2023-01-19

**Authors:** Karin Hallin, Ulrika Larsson, Nadine Schart-Morén

**Affiliations:** Department of Surgical Sciences, Otorhinolaryngology, Uppsala University, 75185 Uppsala, Sweden

**Keywords:** CI, elderly, quality of life, hearing, EQ5D-3L

## Abstract

The present study aims to investigate the usage and benefits of cochlear implants (CIs) in elderly patients aged ≥85 years, including their device-handling issues, follow-ups, and the influence on their well-being. The patients answered one questionnaire regarding quality of life, EQ5D-3L, and one questionnaire, obtained from the Swedish CI quality register, regarding usage, handling, satisfaction, remaining difficulties, etc. The medical records were searched for the implantation date, implant model, speech processor model, monosyllabic (MS) word scores, infections over the implant, and compliance regarding scheduled visits to the clinic. The results show that most elderly patients are satisfied full-time users of their implants. Even though most patients had no problems handling their CI, handling issues must be considered. Recurring guidance and training on device operation are needed. We suggest that follow-up visits are essentially needed for this group of patients on a regular basis. CI surgery is considered a safe treatment, even for the elderly. Upgrads to new external equipment (e.g., sound processors) should not be excluded because of their age. The results suggested that the CI positively affected their well-being. This study was approved by the Swedish Ethical Review Authority (5/10-2021, Dnr: 2021-04970).

## 1. Introduction

The population in the world grows older, and age-related hearing loss is known to substantially impact quality of life [1,2]. Studies have found that the incidence of dementia is higher in people with self-reported hearing problems compared to hearing aid users or cochlear implant users [3]. Age-related hearing loss arises in most people, and it is a known fact that it can substantially impact quality of life and is an independent risk factor for the development of cognitive decline and even Alzheimer’s disease [4,5,6]. Studies estimate that hearing loss may account for 9% of the risk for Alzheimer’s disease [3]. A 25-year prospective study of 3777 people aged 65 years or older found a higher incidence of dementia in those with self-reported hearing problems compared to hearing aid users [7]. Similarly, a cross-sectional study found that persons with untreated hearing who did not use hearing aids showed worse results in cognition tests compared to normal-hearing persons. The study also indicated that social isolation is a crucial mediating factor [8]. A US nationally representative survey tested persons over 50 every two years for 18 years. They found that hearing aid use was positively associated with episodic memory scores. Deterioration in episodic memory scores was slower after than before the use of a hearing aid [9].

Cochlear implantation (CI) is a routine treatment for severe hearing loss. Earlier studies have shown a significant increase in speech perception after CI for all age groups [10,11]. There is no agreement among studies on what defines an elderly patient. Some studies consider those over 65 years to be elderly [12]. Other studies define patients above 60 years as being elderly [13,14]. We have many patients aged 85 and older who are enrolled at our clinic, and we saw the need for a thorough investigation and analysis of this group.

The present study aims to investigate the usage and benefits of CIs in elderly patients aged ≥85 years. Aspects including the handling of the CI processor, if they can come to the clinic if needed, and how the CI has influenced their well-being will be analyzed.

## 2. Materials and Methods

All patients included in this study were 85 years of age and older. They were implanted at Akademiska University Hospital, Uppsala, Sweden or another clinic but did their follow-up checks at Akademiska University Hospital. The patients had implants from Cochlear (Lane Cove, NSW, Australia) and MedEl (Innsbruck, Austria). The patients answered two questionnaires: EQ5D-3L [15] “Reprinted with permission from Ref. [16]. Registration ID: 48413.” and a questionnaire obtained from the Swedish CI quality register [17] that included questions about usage, handling, satisfaction, difficulties, etc. In EQ5D-3L, the patient rated his or her current health status on a scale from 0 to 100. From the answers given by the patient on the five questions in EQ5D-3L, an index value was calculated. The index value is a score between 0 (dead) and 1 (full health). The medical records were searched for the implantation date, implant model, speech processor model, monosyllabic word (MS-word) scores measured with CI only at 65 dB SPL in a sound-treated booth in free field, infections over the implant, and if they had appointments to the clinic that had been canceled for health-related issues. A one-sample Wilcoxon signed rank test (for the non-normally distributed data) was used to calculate if the mean value (VAS and index value from EQ5D-3L) from this study differed significantly (α = 0.05) from the population mean.

All patients aged ≥85 years who were enrolled at our clinic were asked to participate (71 patients). Information about the study, a consent form, and the two questionnaires were sent to the patients via mail, with one reminder if they did not reply. If they were willing to participate, they returned the questionnaires to the clinic via mail, together with the consent form.

## 3. Results

Forty-three people responded (19 males and 24 females). The mean age for the respondents was 88 years, ranging from 85–97. They were implanted at a mean age of 79 years, ranging from 67–90.

**Figure 1:**
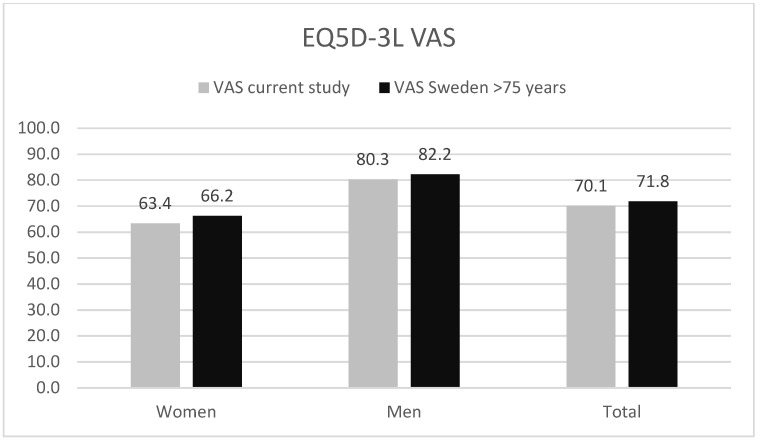
VAS from EQ5D-3L from this study and for an average Swedish population aged 75 and above [18]. The results are displayed in total and separated for men and women.

In Figure 1, the results for this study from the EQ VAS (self-rated health) from the EQ5D-3L are displayed (*n* = 40, three respondents did not answer) together with the mean VAS from an average Swedish population aged 75 and above [18]. No statistically significant difference was found between the current study and the population mean.

**Figure 2:**
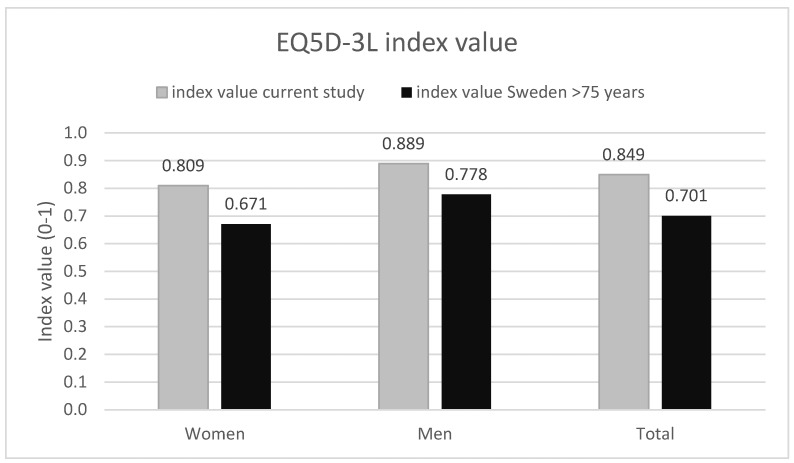
Index value from EQ5D-3L from this study and for an average Swedish population aged 75 and above [18]. The results are displayed in total and separated for men and women.

Figure 2 shows the EQ5D-3L index values for this study (*n* = 38, five did not answer all questions and were excluded) together with the mean index value from the average Swedish population aged 75 and above [18]. For all groups (women, men, and total) there is a significant difference in the index values between the current study and the mean population (*p* < 0.0001).

**Figure 3:**
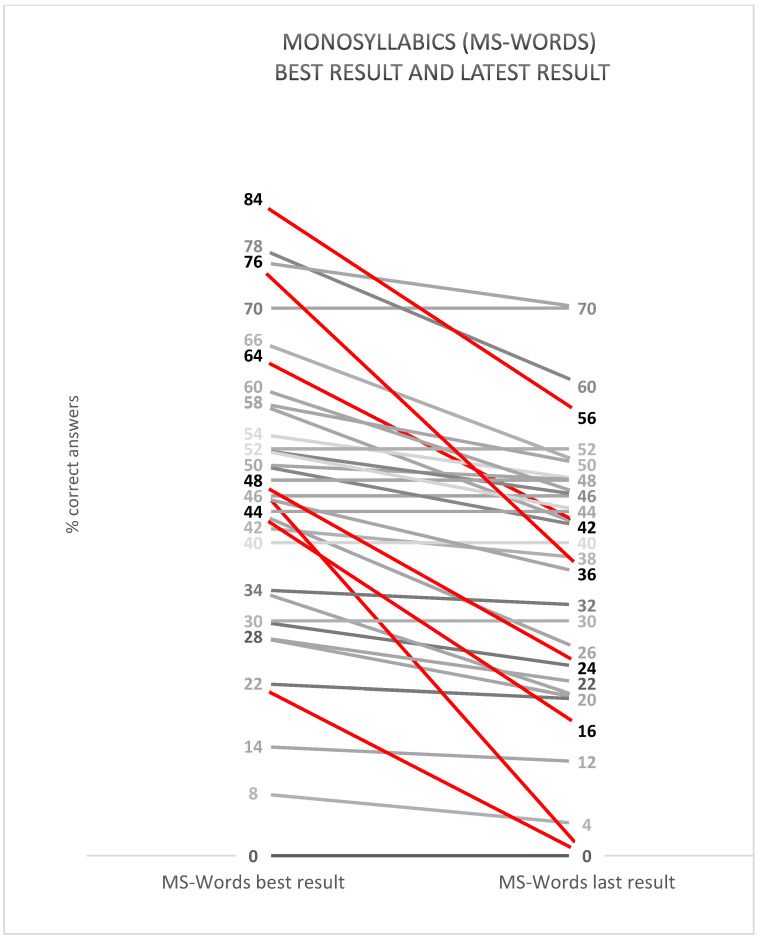
MS words best score and MS words at the latest visit to the clinic for all patients in the study. Patients marked with a red line have a decline in the score of more than 20 percentage points.

Figure 3 shows all patients’ best MS word scores and their latest MS word scores with CI only. The measurements are conducted in a sound-treated booth in a free field at 65 dB SPL. The red lines are the patients with a decline in the MS word score of more than 20 percentage points. Those patients are displayed in more detail in Table 1. The mean value for the best MS word score for the patients in this study was 44%, and for the latest MS word score it was 35%.

Four patients out of the forty-three included in the study canceled appointments at the clinic due to health-related issues that made it difficult to travel to the clinic (3) and the severe sickness of the patient’s spouse (1). None of those four are among the patients with a change in the MSword score of more than 20 percentage points, as shown in Table 1. One patient has had a documented incident of infection over the implant housing but is not one of the patients in Table 1 with a decline in the MS word score of more than 20 percentage points. Five of the forty-three patients in the study live at home with home care or at a nursing home. In 7 of the 43 cases, we do not know their living situation. The remaining 31 patients live at home with no support. One patient living in a nursing home is patient Q41 in Table 1.

**Figure 4:**
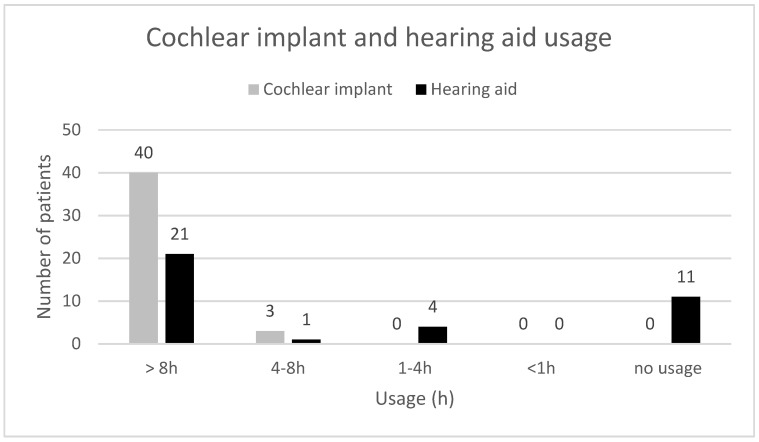
Cochlear implant and hearing aid usage.

**Figure 5:**
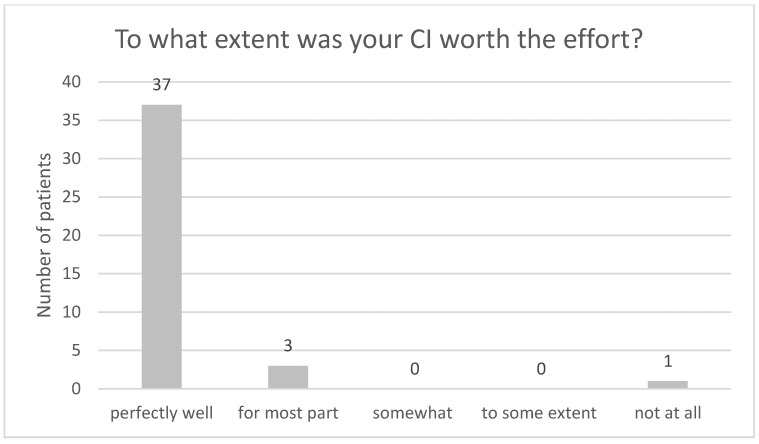
To what extent was your CI worth the effort?

**Figure 6:**
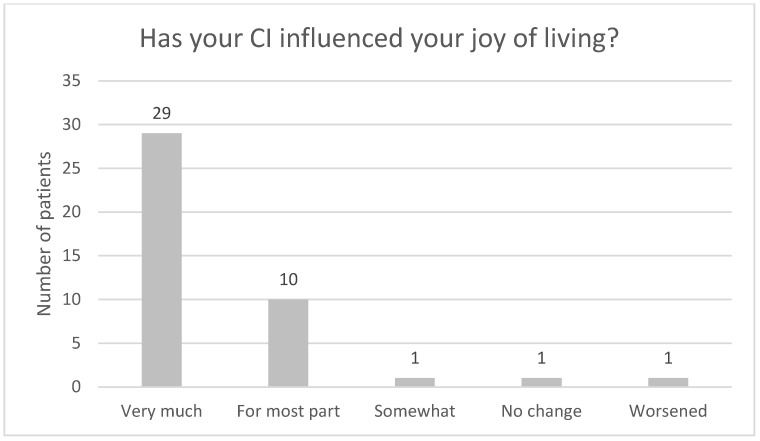
How has your CI influenced your joy of living?

**Figure 7:**
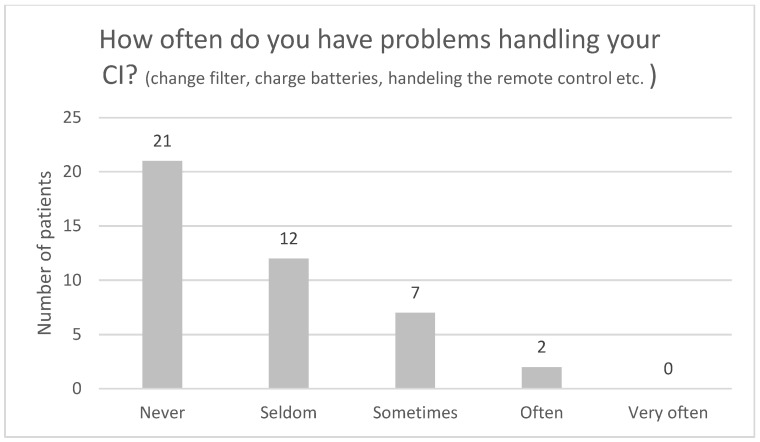
How often do you have problems handling your CI?

**Figure 8:**
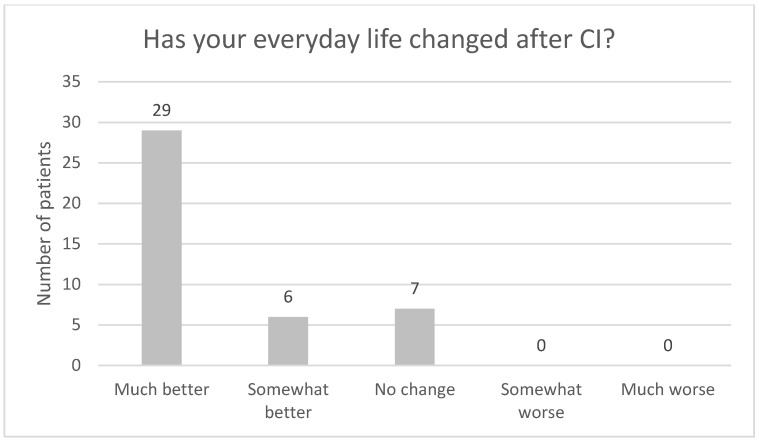
Has your everyday life changed after CI?

**Figure 9:**
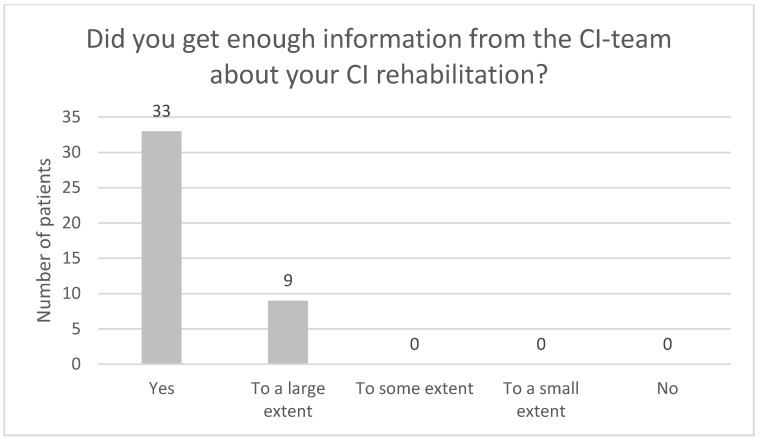
Did you obtain enough information from the CI team about your rehabilitation?

**Figure 10:**
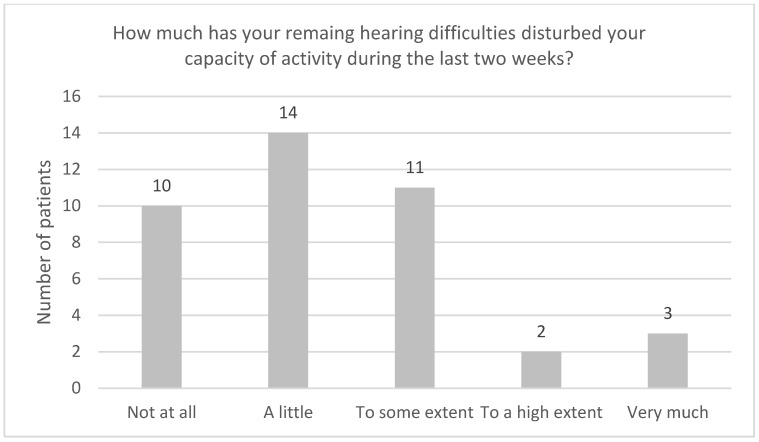
How much have your remaining hearing difficulties disturbed your activity capacity during the last two weeks?

Figure 4, Figure 5, Figure 6, Figure 7, Figure 8, Figure 9 and Figure 10 show a selection of the questions from the Swedish CI quality register questionnaire. The number of patients who have answered the questions is displayed in each figure. Figure 4 shows the CI and hearing aid (HA) usage. Figure 5 shows to what extent patients thought that CI was worth the effort. Figure 6 shows if CI has influenced their joy of living. Figure 7 shows how often they have problems handling their CI. Figure 8 shows if CI has changed their everyday life. Figure 9 shows if they obtained enough information from the CI team about their rehabilitation. Figure 10 shows how much their remaining hearing difficulties disturbed their activity capacity during the last two weeks.

**Figure 11:**
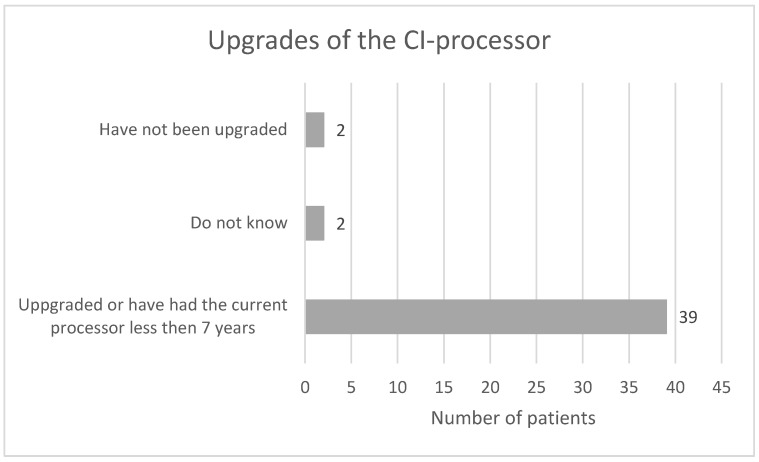
Upgrades of the CI-processor for patients in the study.

Figure 11 shows the number of upgrades of the CI-processor among patients in this study. We have used a seven-year time limit for upgrades at our clinic for all ages. The upgrade status is unclear for two patients, and two patients have not been upgraded. The reason for this was health-related issues that made it hard to travel to the clinic in one case and severe vision problems that made handling a new device hard in another case. None of the non-upgraded patients are among the patients in Table 1.

## 4. Discussion

In this study, CI users showed no significant difference in VAS scores from EQ5D-3L when compared to the reference group from an average Swedish population aged 75 and above [8]. Said de Angelo et al. [19] saw no difference when comparing the QoL for CI users and normal hearing subjects using the World Health Organization Quality of Life (WHOQOL-BREF) generic assessment questionnaire, even though their cohort was extensively younger. The index value from EQ5D-3L shows that our investigated group of CI patients scores significantly higher than the reference group from an average Swedish population aged 75 and above [18].

Hilly et al. [20] saw that cochlear implantation improved audiometric outcomes and quality of life in elderly patients and that these benefits were stable over time. They used Short Form 36 Health Survey questionnaires for evaluation. Saraça et al. [21], on the other hand, found in their study that the QoL of healthy adults was better than that of CI users. They investigated a younger group of patients (mean age just above 40 for both groups) using WHOQOL-BREF.

The seven patients in this study with a decline in speech performance (i.e., a decline in the MS word score of more than 20 percentage points) have been carefully investigated. One of them (Q38) still scores above average compared with all adult patients at our clinic (average MS word score of 47% for adults and 38% for those implanted at age 79 or older [11]). Q19 can no longer handle his or her usual activities but does not score significantly lower on EQ5D-3L than the average Swedish population aged 75 and above. Q29, Q38, and Q41 all are 90 years old or older. We have not found a shared reason for their decline in speech performance.

Almost all the patients in this study used their CI for more than 8 h daily. None of the patients used it for less than 4 h a day. One pitfall in our study is that we do not know the non-participants in this study. One might assume that these individuals might use their CIs less. Thirty-seven out of forty-one patients reported that the CI was worth the effort, and thirty-nine out of forty-two reported that their CI had influenced their joy of living very much or for the most part. Twenty-nine of forty-two reported that their life has become much better after CI. All patients reported that they obtained information from the CI team about the rehabilitation process, at least to a large extent. Overall, the patients were very satisfied with their CI, even though 30 of 40 patients reported that their remaining hearing difficulties still disturbed their activity capacity during the last two weeks. Imagawa et al. [22] found in their study of satisfaction levels among CI users, comparing those over 75 with those under 75 years of age, that the elderly group was less satisfied with their CIs than the younger group, even though 93.3% of all subjects responded that they were “somewhat satisfied” or better. The factors that significantly influenced the satisfaction level were improvements in their ability to understand everyday conversations with family members and to have conversations at reception desks, such as those in banks and shops.

Thirty-three of forty-two patients in the current study have never or seldom had difficulties handling their CI. Two respondents answered that they often have problems. Elderly CI users in the study by Imagawa et al. [22] were highly independent, with basic operations such as attaching, turning on and off, charging batteries, exchanging batteries, and storing the device. However, they were less independent in more complex operations such as adjusting the volume, switching between programs, and exchanging cables. The routine at our clinic is to meet all elderly CI patients (above 85) at least once a year for an audiological and technical evaluation. We give guidance and training on the device operation. This practice adheres to the latest guidelines in Sweden regarding the care of people with severe hearing loss [23]. Our clinic has no age limit for upgrades of the CI processor, since we believe that all ages can benefit from newer techniques. Exceptions can be made for patients with severe illness or dementia because programming a new device requires several clinic visits and the ability to learn how to handle it. However, with exhaustive family and healthcare personnel support, even those patients can benefit from a new device.

In their study comparing speech results, complications, and rehabilitation between people over and under 70 years of age, Rohloff et al. [24] found that the recovery period of vestibular dysfunction after surgery may be longer in the elderly. Otherwise, they saw no difference between groups. An earlier study from our clinic [11] found no severe pre- or postoperative surgical complications among patients who were 79 years old or older at CI surgery.

Raymond et al. [25] found that older geriatric adults do not have higher rates of postoperative healthcare utilization after cochlear implantation than their younger, hearing-impaired counterparts, despite presumed higher rates of frailty and comorbidity.

One concern for older patients is that the surgical procedure with cochlear implantation might have a higher risk for morbidity and mortality or that the aging brain might be more vulnerable to anesthesia. The procedure takes 1.5–2 h and is usually performed under general anesthesia. In some clinics, attempts have been made to do it under local anesthesia [26,27]. However, anesthesia-related complications seem to be rare. Many adverse outcomes may be multifactorial. Postoperative complications are mostly related to the perioperative procedure, not the anesthesia itself [28]. Cochlear implantation is considered a safe treatment with low risks of complications [29].

In summary, hearing loss might result in cognitive decline through reduced cognitive stimulation. Hearing aids may have a modifying effect on the course of cognitive decline in older age. Providing hearing aids or other rehabilitative services for hearing impairment early in life may slow down the growing worldwide incidence of dementia.

## 5. Conclusions

Elderly patients over 85 years old often use their CIs full-time and are mostly satisfied with them. The results from EQ5D-3L suggest that the CI positively affected their well-being. Handling issues must be considered, and reoccurring guidance and training on device operation are needed. Follow-up for this group of patients is essentially needed on a regular basis. CI surgery is considered a safe treatment, even for the elderly. Upgraders to new external equipment (e.g., sound processors) should not be excluded because of age, as a good rehabilitation for hearing loss may slow down the growing worldwide incidence of dementia.

## Figures and Tables

**Table 1:** Patients with a change in monosyllabic score of more than 20 percentage points between best and latest measure, their daily CI usage, EQ5D VAS score, index value (NA = no answer), age, and sex.

Patient	MS Best (%)	MS Latest (%)	Change (Percentage Points)	Usage/Dag (h)	EQ5D VAS	Index Value	Age	Sex	Comment
Q3	64	42	−22	>8 h	80	0.9349	85	W	
Q19	22	0	−22	4–8 h	70	0.7139	88	W	Reported “I cannot handle my usual activities” on EQ-5D. Other health-related issues than hearing difficulties.
Q29	48	0	−48	>8 h	90	0.9694	90	W	
Q38	84	56	−28	>8 h	70	0.9694	97	M	Even after a significant decline in results, these results are better than average for adults at our clinic.
Q40	48	24	−24	>8 h	72	NA	88	W	The patient has been upgraded since the last MS word test. The best MS word score is measured on a higher presentation level than the latest measure.
Q41	44	16	−28	>8 h	NA	NA	96	M	The patient lives at a nursing home. Vision impaired. Isolated.
Q42	76	36	−40	>8 h	80	0.9349	85	M	

## Data Availability

Not applicable.
